# A multifunctional EGCG/Si nanohybrid-coated 3D-printed porous scaffold for bone defect repair

**DOI:** 10.1093/rb/rbag035

**Published:** 2026-03-09

**Authors:** Shiqi Xiao, Lin Qi, Jiacheng Wei, Huan Liu, Sheng Ding, Ju Chen, Jin Yang, Hua Lin, Dianxiang Lu

**Affiliations:** Clinical Medical College & Affiliated Hospital of Chengdu University, Chengdu University, Chengdu 610081, China; Research Center for Nano-Biomaterials, Analytical and Testing Center, Sichuan University, Chengdu 610064, China; Department of Criminal Science and Technology, Sichuan Police College, Luzhou 646000, China; School of Clinical Medicine, North Sichuan Medical College, Nanchong 637000, China; School of Clinical Medicine, North Sichuan Medical College, Nanchong 637000, China; Clinical Medical College & Affiliated Hospital of Chengdu University, Chengdu University, Chengdu 610081, China; Clinical Medical College & Affiliated Hospital of Chengdu University, Chengdu University, Chengdu 610081, China; Clinical Medical College & Affiliated Hospital of Chengdu University, Chengdu University, Chengdu 610081, China; Department of Stomatology, The First Affiliated Hospital of Chengdu Medical College, Chengdu 610500, China; Clinical Medical College & Affiliated Hospital of Chengdu University, Chengdu University, Chengdu 610081, China

**Keywords:** bone regeneration, EGCG/Si nanohybrid coating, 3D printing, multifunctional scaffolds

## Abstract

Besides enhancing osteogenesis and angiogenesis, designing multifunctional scaffolds with immunomodulatory capabilities offers a promising strategy for managing bone defects. Herein, a 3D-printed scaffold modified with inorganic silicon (Si) ions and the polyphenol epigallocatechin gallate (EGCG) was constructed as an immunomodulatory biocomposite with coupled angiogenic and osteogenic activity to enhance bone regeneration. Through phenol-amine chemistry and electrostatic layer-by-layer deposition, a nanohybrid EGCG/Si coating was fabricated on the poly(lactic-co-glycolic acid) scaffold surface. Our *in vitro* studies demonstrated that the released Si ions, in combination with the surface topological morphology, promoted osteogenic differentiation of bone mesenchymal stem cells and enhanced the ability of human umbilical endothelial cells to form patterned vascular networks. The EGCG-coated scaffold not only provides effective protection against reactive oxygen species-mediated cellular damage but also actively modulates the immune microenvironment by inducing macrophage polarization toward a reparative phenotype, enhancing the expression of anti-inflammatory factors and inhibiting pro-inflammatory gene expression. *In vivo* experiments further revealed that the coated scaffolds exhibited significant potential in enhancing new bone formation within rat femoral defects. Transcriptomic profiling indicated that Si ions, in conjunction with surface topography, cooperatively triggered multiple signaling pathways involved in cell adhesion, proliferation and differentiation. Overall, this EGCG/Si nanocomposite coating approach presents a novel avenue for developing multifunctional scaffolds in bone tissue engineering.

## Graphical abstract

**Graphical Abstract:**
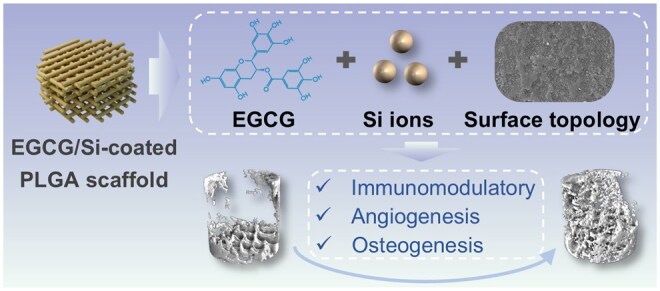

## Introduction

Bone defects represent a prevalent and challenging medical issue in clinical practice. The development of biomaterials that combine favorable bone repair efficacy with cost-effectiveness is highly important clinically managing bone defects [1]. In recent years, tissue engineered scaffolds have been increasingly applied in bone defect repair. Among various fabrication techniques, 3D printing has emerged as one of the leading methods for constructing bone tissue engineering scaffolds, owing to its ability to precisely control scaffold shape, pore architecture and internal interconnectivity [2, 3]. The obtained porous scaffolds create a conducive microenvironment that supports critical biological events, encompassing cell spreading, vascularization and osteogenesis [4, 5]. Benefiting from excellent biocompatibility and controllable degradation profile, the synthetic copolymer poly(lactic-co-glycolic acid) (PLGA) is widely utilized in fabricating 3D-printed scaffolds for bone tissue engineering [6]. However, the inherent hydrophobicity of PLGA and its lack of cell-recognition motifs on the surface constrain the regulation of early cellular behaviors and subsequent bone regeneration efficacy [7].

In bone tissue engineering, early interactions between implanted materials and host cells primarily occur at the material surface, making surface modification strategies a focus of extensive research. The incorporation of bioactive inorganic elements, including silicon (Si) [8], magnesium [9], calcium [10], zinc [11] and copper [12] ions, has become an important approach to enhance the biological performance of materials. Among these, Si ions demonstrate osteoinductive capabilities as well as the capacity to form direct bonds with both hard and soft tissues. This phenomenon is due to the interaction of silanol groups with calcium and phosphate ions in physiological environments, thereby promoting the nucleation and growth of a carbonated hydroxyapatite layer [13], which enhances bone regeneration. Moreover, the pro-angiogenic effect of silicon offers an additional advantage for bone tissue regeneration [14]. Since the host tissue identifies the implant as a foreign body, the placement of exogenous biomaterials into bone defects often induces inflammatory reactions. Extensive research indicates that the initial immune cell response to the material surface, regulated via immunomodulatory mechanisms, plays a decisive role in the implant’s long-term outcome, occurring prior to the onset of angiogenesis and osteogenesis [15]. Thus, beyond their osteogenic and angiogenic capabilities, immunomodulatory functions of biomaterials are equally essential in the development of bone regeneration scaffolds.

Epigallocatechin gallate (EGCG), a green tea-derived polyphenol, possesses a molecular architecture rich in phenolic hydroxyl groups. This structure grants it significant antioxidant capacity, allowing it to neutralize reactive oxygen and nitrogen species effectively, thereby alleviating the inflammatory state in the injured area [16, 17]. Notably, the phenolic hydroxyl structure of EGCG endows it with broad-spectrum adhesion properties. Based on this, researchers often employ a simple one-pot reaction to apply EGCG for the surface modification of tissue engineering scaffolds [18, 19]. This approach not only directly enhances the surface bioactivity of the scaffold but, more importantly, allows EGCG to act as an efficient bio-mediator. Through its polyphenolic groups, EGCG can undergo coupling reactions with other bioactive components such as metal ions [20] and proteins [21], thereby firmly anchoring these functional molecules onto the material surface and constructing a multifunctional biointerface. It has been reported that phenol-amine reactions can occur [22], and amine groups become positively charged upon protonation. Therefore, we propose the following hypothesis: first, the phenol-amine complex is preferentially deposited on the scaffold surface, imparting a positive charge; subsequently, negatively charged silicate ions are further deposited on the material surface via electrostatic interactions, ultimately forming an organic/inorganic hybrid coating enriched with both polyphenols and silicon. This coating is expected to integrate the biological functions of polyphenols with the osteogenic activity of Si ions, offering a novel approach for designing functionally enhanced surfaces in bone repair materials.

This study presents a multifunctional 3D-printed scaffold with immunomodulatory, osteogenic and angiogenesis-enhancing capabilities. A porous PLGA scaffold was created using 3D printing to establish a 3D environment that supports cell proliferation and tissue ingrowth. Subsequently, leveraging the strong adhesion of polyphenols, EGCG was deposited onto the surface of the PLGA scaffold along with polyethylenimine (PEI). The opposing charges of the phenol-amine coating (positive) and the silicate ions (negative) enabled the successful assembly of a Si-based layer on the scaffold surface through electrostatic forces. By repeating this deposition process, a 3D porous PLGA composite scaffold featuring a layered architecture composed of silica and polyphenol elements was successfully constructed. In this system, EGCG not only acts as a bridging molecule to firmly anchor the Si coating onto the scaffold surface but also effectively modulates the implant site microenvironment through its inherent antioxidant and anti-inflammatory activities. By combining the biological activities of EGCG with the dual functions of Si ions in osteogenesis and angiogenesis, this composite scaffold exhibits promising capabilities for cooperatively enhancing bone defect regeneration.

## Experimental

### Materials

PLGA (with an 85:15 LA/GA ratio and a molecular weight of 300 kDa) and dichloromethane were obtained to fabricate 3D-printed porous scaffolds. Tetramethyl orthosilicate, EGCG and PEI were acquired to prepare EGCG/Si nanohybrid coating. A comprehensive list of the materials and instruments utilized in this work can be found in Tables S1 and S2.

### Preparation of 3D printed scaffolds

The PLGA scaffold’s matrix architecture was constructed using a commercial 3D bioprinter through a dissolved deposition modeling technique. Before printing, a 25% (w/v) PLGA ink formulation was synthesized through dissolution of the polymer in dichloromethane under constant agitation. The mixture was stirred until it achieved an appropriate viscosity for extrusion-based printing, after which it was loaded into the printer’s material cartridge. Printing utilized a 0.28 mm nozzle diameter, 0.7 mm filament spacing, 0.2 mm layer height and a 0°/90° cross-hatching deposition pattern. Following fabrication, the printed scaffolds were subjected to vacuum drying for 1 week to ensure complete evaporation of any remaining solvent.

### Preparation of EGCG/Si coating

The nanohybrid EGCG/Si coating was applied to the PLGA scaffolds via a layer-by-layer deposition. First, coating solutions were prepared by dissolving EGCG and PEI in Tris buffer adjusted to pH 8.5, with the EGCG to PEI concentration ratios set at 2:1 and 1:2. Following a 4-h submersion in these solutions, the PLGA scaffolds were thoroughly rinsed with distilled water and dried to eliminate loosely bound molecules. For the silicon deposition process, a 1 M silicic acid solution was prepared via the acid-catalysed hydrolysis of tetramethyl orthosilicate in HCl. The scaffolds coated with EGCG and PEI were then immersed in this pre-hydrolysed tetramethyl orthosilicate solution for 4 h, after which they were cleaned and dried. This cycle of immersion in EGCG/PEI solution and subsequent silicification was repeated ten times. The resulting samples were designated as nSC1 and nSC2, corresponding to the initial EGCG:PEI molar ratios of 2:1 and 1:2, respectively. Furthermore, to elucidate the role of EGCG/PEI assemblies to coating formation, specimens designated as E/P_1_, E/P_2_ and E/P_2_-Si were prepared for characterization via XPS and zeta potential measurements. The E/P_1_ and E/P_2_ scaffolds were coated with a single EGCG/PEI layer at molar ratios of 2:1 and 1:2, respectively, while the E/P_2_-Si scaffold featured an additional silicified layer deposited atop the E/P_2_ surface.

### Characterization of EGCG/Si-coated scaffolds

The surface morphologies of the coated scaffolds were examined using SEM, with elemental analysis performed by the integrated EDS system. The chemical structure of the coating was characterized by XPS. The wettability of the surface was determined by static sessile drop water contact angle measurements. The surface potential at pH 3 was determined using a Zeta potential analyser. The morphology of the HA was characterized by TEM. The UV–vis absorption of the EGCG/PEI coating solutions was measured with a UV–vis spectrophotometer. The Si ion release profile was monitored by immersing the scaffolds in 4 mL of PBS (pH 7.4) maintained at 37°C. The supernatant was sampled at designated intervals, and the Si ions concentration was measured via ICP-OES. The phenolic content present on the scaffold surface was assessed using the Folin–Ciocalteu assay. In brief, EGCG/Si-coated scaffolds were immersed 6.25% in Folin–Ciocalteu solution for 10 min. Subsequently, a 20% w/v sodium carbonate solution was introduced. The reaction system was kept at room temperature for 2 h, after which the absorbance was recorded at 780 nm with a multilabel plate reader.

### Viability and osteogenesis evaluation of BMSCs

BMSCs were extracted from Sprague-Dawley rats aged 3 weeks and maintained in α-MEM supplemented with 1% Pen Strep and 10% NBCS. SEM was employed to evaluate the adhesion of BMSCs on the scaffold surfaces. The proliferation of BMSCs on the scaffolds was assessed via CCK-8 assay at 1, 3 and 5 days. To evaluate the intrinsic osteoinductive potential of the EGCG/Si-coated scaffolds, osteogenic differentiation was induced in the absence of exogenous factors (e.g. dexamethasone or BMP-2); the medium was supplemented solely with 10 μg mL^−1^ ascorbic acid and 10 mM β-glycerophosphate. ALP activity was quantified after 7, 14 and 21 days using an ALP Assay Kit. Calcium deposition was evaluated using ARS staining on day 21. Additionally, the gene expression levels of osteogenic markers (*ALP*, *COL1*, *OCN*, *OPN*, *Osterix* and *Runx2*) were analysed by RT-PCR after 7 days of culture, with the primers sequence listed in Table S3. Detailed experimental methods can be found in Text S1 and S2.

### Antioxidant evaluation

#### DPPH radical scavenging assay

The antioxidant ability of the scaffolds was initially assessed by the DPPH method. In brief, each scaffold was immersed in 3 mL DPPH solution and incubated in darkness for 30 min. Following incubation, the absorbance of the supernatant was recorded at 515 nm with a microplate reader, with pure DPPH solution serving as the blank. The scavenging activity was thereby determined using the equation below:


(1) $$\mathrm{DPPH}\;\mathrm{scavenging}\;(\%)=\frac{A_{\mathrm{blank}}-A_{\mathrm{scaffolds}}}{A_{\mathrm{blank}}}\times 100\%$$

where *A*_scaffolds_ and *A*_blank_ represents the absorbance of the DPPH solution in the presence and absence of the scaffold, respectively.

#### Cytoprotective effects against exogenous H_2_O_2_

BMSCs were seeded onto both PLGA and EGCG/Si-coated PLGA scaffolds, with cells on culture plates as a control. Following a 24-h adhesion period, cells were exposed to either 0.2 mM H_2_O_2_ or left untreated for 12 h. Cell viability was subsequently determined using the CCK-8 assay.

#### Intracellular ROS scavenging assessment

A ROS Assay Kit was employed to evaluate the intracellular ROS-scavenging capacity of the scaffolds. BMSCs were cultured in 24-well plates at 5 × 10^4^ cells per well. Following a 24-h incubation period, BMSCs were exposed to 0.5 mL of DCFH-DA probe (1 μL mL^−1^) in medium without serum for 30 min in the dark to promote intracellular uptake. After three washes with serum-free medium to eliminate excess probe, cells were exposed to Rosup (1 µg mL^−1^) with or without scaffolds for 30 min. Only rosup-treated cells and untreated cells were designated as the positive and negative controls, respectively. ROS-generated fluorescence was visualized using a fluorescence microscope.

### Angiogenesis evaluation

#### Cell migration assay

A scratch wound healing assay was conducted to evaluate the migratory behavior of HUVECs. Briefly, cells were plated in 24-well plates at 5 × 10^4^ cells per well and grown until a confluent monolayer was established. Subsequently, a standardized wound was introduced into each monolayer by scraping with a sterile 200 μL pipette tip. Subsequently, dislodged cells were removed through three PBS washes, after which cells were replenished with conditioned medium. This medium was obtained by incubating the scaffolds in high-glucose DMEM for 24 h. Wound healing was documented at 0, 6 and 24 h post-scratching using an inverted microscope. Migration ratio was calculated using the formula:


(2) $$\mathrm{Migration}\;\mathrm{ratio}\;(\%)=\frac{\mathrm{Original}\;\mathrm{sractch}\;\mathrm{area}-\mathrm{Finial}\;\mathrm{scratch}\;\mathrm{area}}{\mathrm{Original}\;\mathrm{scratch}\;\mathrm{area}}\times 100\%$$

#### Tube formation assay of HUVECs

The tube-forming capability of HUVECs was examined using a Matrigel assay. Matrigel was evenly distributed onto culture plates and maintained at 37°C for 30 min to promote solidification of the matrix. Subsequently, cells were inoculated onto the pre-coated matrix at 5 × 10^4^ cells per well and incubated in conditioned medium for 8 h. Capillary-like networks were visualized under an inverted light microscope, and representative images were recorded for quantitative assessment. We measured tubular structure parameters such as the number of nodes, number of enclosed meshes, total mesh area and cumulative tube length using ImageJ software.

### Immunomodulatory property analysis

The anti-inflammatory activities of the scaffolds were analysed with RAW264.7 macrophages. The cells were plated in 24-well plates at a density of 1 × 10^5^ cells per well, followed by an overnight incubation to ensure cell attachment. To induce an inflammatory response, cells were treated with LPS at a concentration of 500 ng mL^−1^ for 4 h. Next, the cells were maintained in conditioned medium, which was obtained after incubating scaffolds with RPIM medium 1640 for 24 h. Following 24 h of culturing, the mRNA expression levels of pro-inflammatory cytokines *TNF-α* and *IL-1β*, along with anti-inflammatory markers *IL-10* and *Arg-1*, were quantified using RT-PCR. Relative gene expression, analysed by the 2^−ΔΔ^^*Ct*^ method and normalized to the negative control (cells not exposed to LPS), is presented with corresponding primer sequences in Table S3. Immunofluorescence staining for iNOS (an M1 marker) and CD206 (an M2 marker) was performed to further assess macrophage polarization. Fluorescent signals were captured using a CLSM.

### Transcriptome sequencing analysis

Following a 7-day culture of BMSCs on nSC2 scaffolds, total RNA was then extracted with TRIzol reagent, and samples were stored at −80°C until further processing. Transcriptome sequencing was performed using the Illumina NovaSeq 6000 platform. Gene expression levels were normalized and reported as transcripts per million (TPM). Differential expression analysis was conducted using DESeq2, identifying significantly differentially expressed genes (DEGs) with an adjusted *P*-value below 0.05 and a fold change exceeding 2. Finally, functional enrichment analysis for Gene Ontology (GO) terms and Kyoto Encyclopedia of Genes and Genomes (KEGG) pathway was performed via the Majorbio Cloud Platform (www.majorbio.com).

### *In vivo* implantation in a rat femur defect model

All animal studies were performed in accordance with established ethical standards, following a protocol that was reviewed and approved by the Ethics Committee of Sichuan University (20211528A). The bone defect repair model was established using male Sprague-Dawley rats (weighing approximately 300 g). Briefly, after anesthetization with 2.5% pentobarbital sodium, bilateral circular defects (3.5 mm in diameter × 4 mm in depth) were surgically induced in the femoral condyles. The created defects were allocated into three experimental groups: one group was left untreated as a control, while the other two groups received implants of either the nSC2 scaffold or the HA scaffold (PLGA scaffolds incorporated with hydroxyapatite) (*n* = 4 per group). The rats were euthanized after a 4-week implantation period. The femoral condyles were oriented for high-resolution micro-CT using a VivaCT80 system to quantitatively assess newly formed bone tissue. Then, 3D reconstruction was performed to analyse bone parameters such as bone volume fraction (BV/TV), trabecular number (Tb.N) and trabecular spacing (Tb.Sp). Thereafter, the specimens were decalcified using EDTA solution to prepare for histological and immunohistochemical analyses, which included hematoxylin and eosin (H&E) staining and osteocalcin (OCN) immunostaining.

### Statistical analysis

The data are expressed as mean ± standard deviation. Statistical differences were assessed through one-way ANOVA with Tukey’s *post hoc* test for multiple comparisons. A *P*-value below 0.05 was considered indicative of statistical significance.

## Results and discussion

### Characterization of EGCG/Si-coated PLGA scaffold

**Figure 1:**
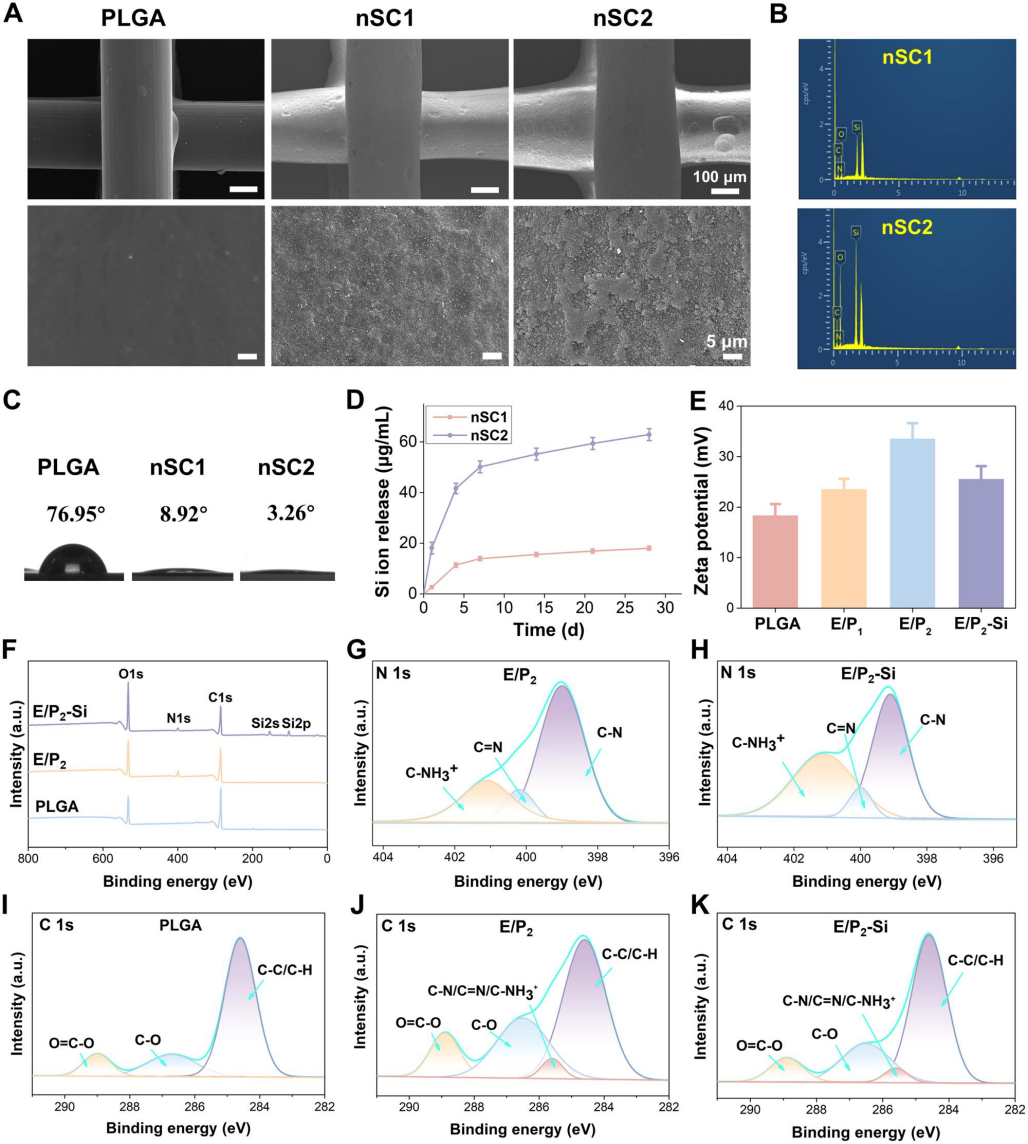
Characterization of the EGCG/Si-coated scaffolds. (**A**) SEM images of PLGA, nSC1 and nSC2 scaffolds. (**B**) Elemental mapping of nSC1 and nSC2 scaffolds. (**C**) Water contact angles of uncoated and coated PLGA scaffolds. (**D**) Release profiles of Si ions from EGCG/Si-coated PLGA scaffolds. (**E**) Zeta potentials of PLGA and coated PLGA scaffolds. (**F**) The XPS spectrums of PLGA, EGCG/PEI-coated PLGA and silica-coated PLGA scaffolds. (**G–K**) High-resolution XPS spectra of N1s and C1s regions.

We first characterized the topographical features and chemical composition of the EGCG/Si-coated scaffolds. As illustrated in Figure 1A, SEM imaging showed that the unmodified PLGA scaffold had a relatively smooth surface, while the EGCG/Si-modified scaffolds displayed a markedly rougher topography. EDS revealed a distinct Si peak (Figure 1B), confirming the successful deposition of Si onto the scaffold surface. Notably, the nSC2 scaffolds exhibited a higher Si content compared to the nSC1 group. This finding can be attributed to the higher proportion of PEI in the nSC2 scaffolds; the positively charged PEI facilitates the recruitment of negatively charged silanol groups via electrostatic interactions, thereby resulting in a coating with higher Si density. Additionally, water contact angle measurements (Figure 1C) demonstrated a substantial enhancement in hydrophilicity following coating: the contact angle dropped from 76.95° for pristine PLGA to 8.92° for nSC1 and further to 3.26° for nSC2, indicating a highly hydrophilic surface after modification. The increase in surface roughness, combined with the chemical composition rich in hydrophilic Si–OH groups, collectively contributed to this wettability transition. Although PLGA possesses good biocompatibility, its inherent hydrophobicity and lack of surface bioactive sites limit cell–material interactions, thereby resulting in insufficient bioactivity. By constructing a nanohybrid EGCG/Si coating on the PLGA surface, this study effectively improved its surface properties. It has been reported that rough and hydrophilic surfaces are more conducive to cell adhesion [23]. Si ion release behavior from the coated scaffolds was further evaluated. As shown in Figure 1D, both nSC1 and nSC2 scaffolds exhibited sustained Si ion release over 4 weeks, marked by a burst release during the first week and a gradual reduction in release rate in the subsequent weeks. Due to its higher surface Si content, the nSC2 scaffold showed a higher cumulative release concentration than nSC1. By the end of week 4, the cumulative release concentrations of nSC1 and nSC2 were 18.0 and 62.9 μg mL^−1^, respectively, both values being substantially below the cytotoxic thresholds documented in previous studies [24].

The surface chemistry of the coated scaffolds was characterized by XPS. The survey spectra (Figure 1F) confirmed the introduction of N after EGCG/PEI coating and the appearance of Si following silicification. High-resolution scans provided further insight into the chemical bonding (Figure 1G–K). The PLGA high-resolution C 1s region confirmed a chemical structure comprising C–C/C–H, C–O and O=C–O bonds, evidenced by peaks centered at 284.6, 286.2 and 288.7 eV, respectively. The emergence of a new peak at 285.6 eV in the coated sample, attributable to C–N/C=N/C–${\mathrm{NH}}_{3}^{+}$ species, providing clear evidence for the effective conjugation and integration of EGCG and PEI. In the N 1s spectrum, the peaks at 399.1 eV (C–N), 400.1 eV (C=N) and 401.1 eV (C–${\mathrm{NH}}_{3}^{+}$) were identified. The presence of the C–${\mathrm{NH}}_{3}^{+}$ species may be explained by proton transfer from the catechol to the amine group [25]. The detection of C=N validates the Schiff base reaction between quinones in EGCG and the amine groups in PEI [26]. Furthermore, UV–visible absorption spectroscopy of the EGCG/PEI reaction system (Figure S1) revealed a characteristic absorption peak at 450 nm, providing additional evidence for the formation of a crosslinked structure via Schiff base interactions between the quinone and amine groups [27]. Surface zeta potential of the scaffold was further determined. As shown in Figure 1E, PLGA scaffold without modification exhibited a zeta potential of 18.3 mV. Following the deposition of EGCG/PEI coatings with varying molar ratios, the surface zeta potential shifted positively to 23.4 mV (EGCG: PEI = 2:1) and 33.5 mV (EGCG: PEI = 1:2), respectively. This elevation is attributed to the positive charges introduced by the protonated amine groups inherent in PEI, with higher PEI concentrations correlating to increased surface positivity. Subsequent Si deposition significantly attenuated the zeta potential to 25.5 mV. This reversal confirms the electrostatic attraction between anionic silicic acid precursors and protonated amino groups on the coating, which served as a key mechanism driving the successful deposition of Si coating.

### Antioxidative and anti-inflammatory properties of nanohybrid EGCG/Si coating

**Figure 2:**
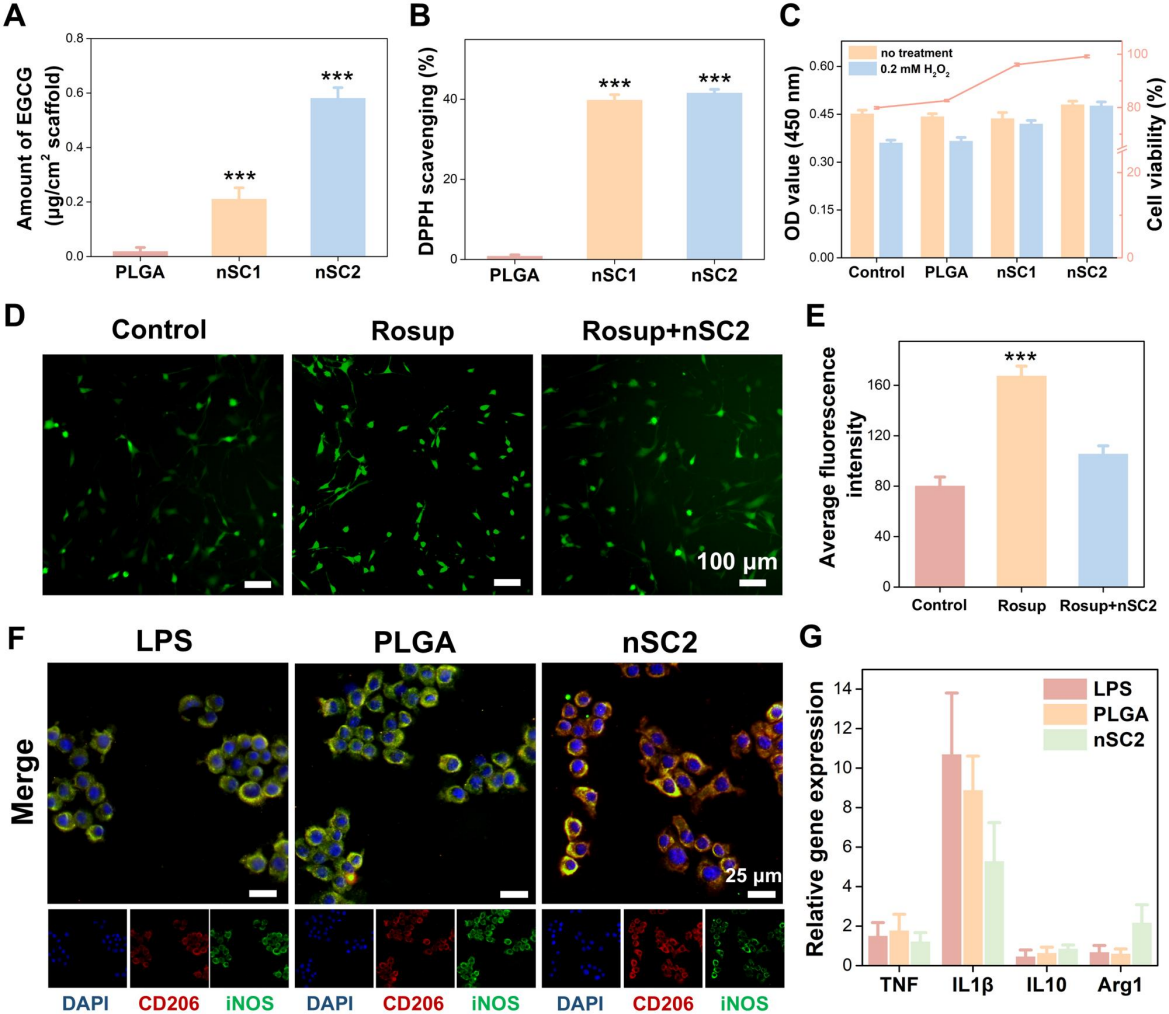
Antioxidative and anti-inflammatory effects of EGCG/Si coating. (**A**) Quantification of phenolic content on the scaffold surface. (**B**) DPPH free radical scavenging ratio. (**C**) Quantitative analysis of BMSCs viability following 12-h culture on scaffolds, both in the presence and absence of 0.2 mM H_2_O_2_ exposure. (**D**) Representative fluorescence images showing intracellular ROS levels in BMSCs incubated with scaffolds following Rosup stimulation. (**E**) Quantification of ROS levels based on fluorescence intensity. (**F**) Representative fluorescence immunostaining images showing iNOS and CD206 expression in RAW264.7 macrophages. (**G**) Inflammatory cytokine mRNA expression levels in RAW 264.7 macrophages. ***P < 0.001 vs control.

The overproduction of ROS at damaged sites disrupts redox homeostasis, creating a pathological microenvironment detrimental to osteogenesis [28]. Therefore, endowing bone repair materials with ROS-scavenging capabilities has emerged as a beneficial strategy to mitigate oxidative damage and proactively support new bone formation. Given the abundant catechol groups in EGCG molecules, the coating fabricated from EGCG is expected to exhibit strong antioxidant properties. The phenolic content on the scaffold surface was first determined using the Folin–Ciocalteu method, where the reagent reacts with phenolic hydroxyl groups to produce a blue coloration, the intensity of which is proportional to the number of phenolic groups. As shown in Figure 2A, significantly higher EGCG content was detected on the surfaces of all coated scaffolds compared to the bare PLGA scaffolds. Furthermore, the nSC2 group exhibited a greater surface phenolic content compared to the nSC1 group. Studies have shown that introducing PEI promotes the formation of phenol-amine aggregates on material surfaces through amine-phenol cross-linking [27]. Consequently, when a lower concentration of PEI (as in the nSC1 group) is cross-linked with EGCG, fewer EGCG/PEI cross-linked structures are formed on the material surface, resulting in lower surface phenolic content compared to the nSC2 group.

We further analysed the free radical scavenging capacity of the coated scaffolds by DPPH method. The results (Figure 2B) demonstrated that both coated scaffolds effectively scavenged free radicals, with the nSC2 scaffold, which had a higher surface phenolic hydroxyl content, exhibiting superior scavenging activity. Subsequently, we assessed the cytoprotective antioxidant efficacy of the scaffolds through a protective assay against H_2_O_2_-induced damage and ROS level detection. As illustrated in Figure 2C, following H_2_O_2_ exposure, the control and PLGA groups exhibited markedly reduced cell viability compared to the nSC1 and nSC2 groups. Measurements of ROS levels (Figure 2D and E) demonstrated that Rosup stimulation significantly elevated intracellular ROS levels, as indicated by increased fluorescence intensity, confirming the successful induction of oxidative stress. In contrast, cells co-cultured with the nSC2 scaffold showed a significant reduction in ROS levels. These findings collectively indicate that the coated scaffolds mitigate H_2_O_2_-mediated oxidative damage by efficiently reducing intracellular ROS levels, thereby enhancing cell viability.

Macrophages play a pivotal role in the immune response to scaffold implantation, polarizing into distinct phenotypes that critically regulate tissue repair. The pro-inflammatory M1 phenotype drives inflammation, while the anti-inflammatory M2 phenotype promotes tissue healing. Therefore, biomaterials capable of modulating macrophage polarization toward the M2 phenotype from the M1 state are more favorable for promoting tissue regeneration [29, 30]. Existing studies have confirmed that EGCG accelerates tissue repair by targeting caspase-1 and NF-κB signaling pathways, thereby suppressing RANKL-mediated inflammatory cascades [31, 32]. To assess the immunomodulatory properties of coated scaffolds on LPS-activated RAW264.7 macrophages, we detected the inflammation-related gene expression and examined the polarization markers iNOS (M1) and CD206 (M2) using immunofluorescence staining. Immunofluorescence staining results (Figure 2F and Figure S2) revealed that the nSC2 group exhibited significantly reduced iNOS expression compared to the LPS and PLGA groups, accompanied by significantly elevated CD206 levels. RT-PCR analysis (Figure 2G) further revealed that, in comparison to the PLGA and LPS groups, the nSC2 group markedly downregulated pro-inflammatory (*TNF-α*, *IL-1β*) and upregulated anti-inflammatory (*IL-10*, *Arg-1*) gene expression. This verifies the dual anti-inflammatory and antioxidant functionality of the EGCG-based nanohybrid coating.

### Effects of the nanohybrid EGCG/Si coating on osteogenesis and angiogenesis

**Figure 3:**
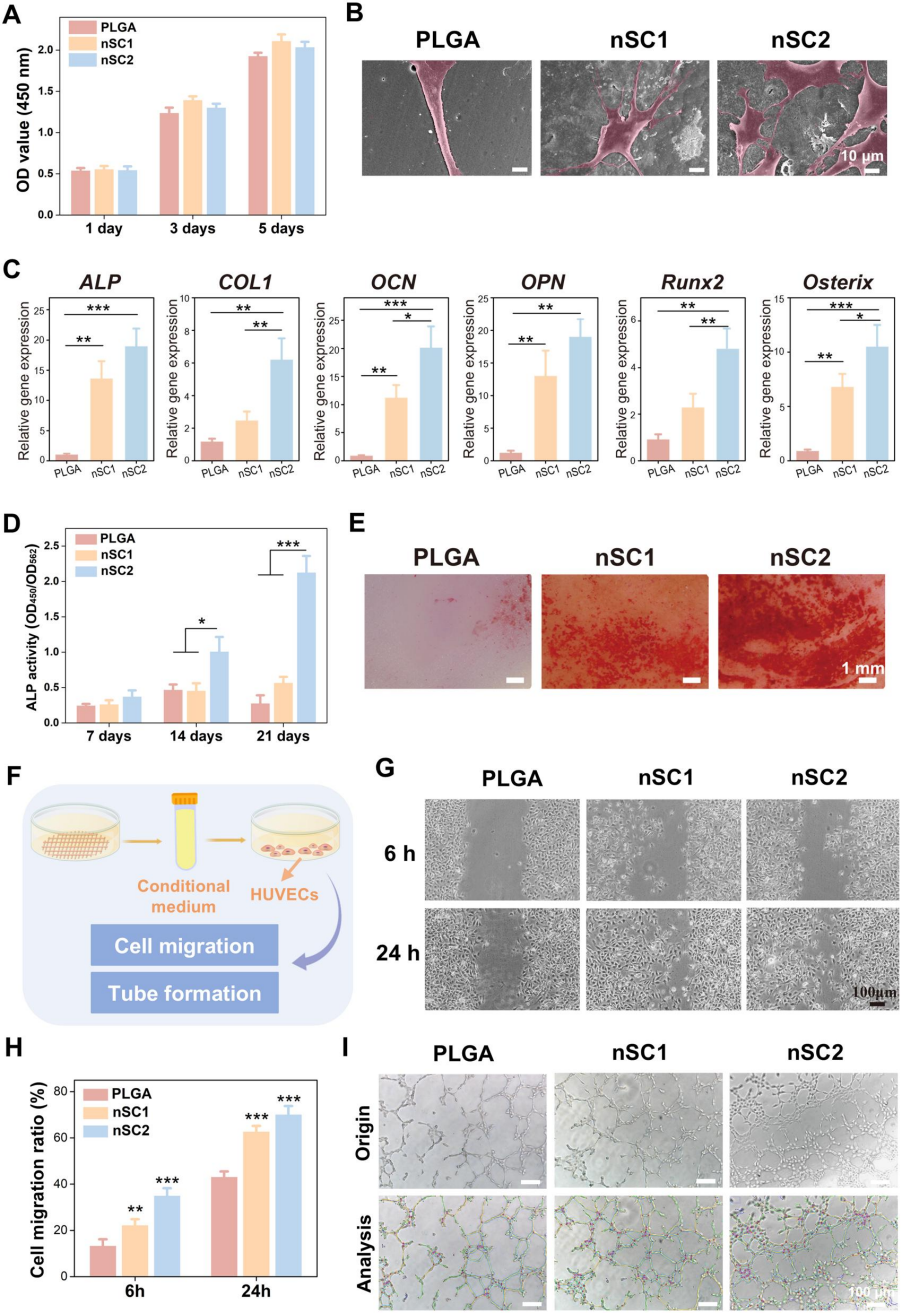
*In vitro* osteogenesis and angiogenesis evaluation. (**A**) Quantitative analysis of cell proliferation using CCK-8 assay after culturing with EGCG/Si-coated scaffolds for 1, 3 and 5 days. (**B**) Representative SEM images of BMSCs cultured on the scaffold surface after 3 days. (**C**) Quantitative analysis of osteogenic-related gene expression (*ALP*, *COL1*, *OCN*, *OPN*, *Runx2* and *Osterix*) determined by RT-PCR. (**D**) Quantitative assessment of ALP activity in BMSCs at 7, 14 and 21 days. (**E**) ARS staining images of BMSCs after 21 days. (**F**) Schematic illustration of the experimental design. (**G**) Representative images showing the migration of HUVECs after incubation with conditioned medium for 6 and 24 h. (**H**) Quantitative analysis of the cell migration rate. (**I**) Representative images of tube formation assay. *P < 0.05, **P < 0.01 and ***P < 0.001 vs control.

The cytocompatibility of the coated scaffolds was assessed using the CCK-8 assay. As illustrated in Figure 3A, the EGCG/Si coating significantly promoted BMSC proliferation compared to the pure PLGA scaffold at days 3 and 5, demonstrating excellent biocompatibility of the modified scaffolds. However, the nSC2 group showed reduced proliferation activity relative to the nSC1 group. This might be due to the higher Si content in nSC2, which corresponds to a greater amount of PEI; the protonated amine groups in PEI, with their high charge density, may exert certain cytotoxic effects [33]. To observe the adhesion morphology, the structural features of BMSCs attached to the scaffold surfaces were further examined (Figure 3B). Cells cultured on the nSC1 and nSC2 scaffolds displayed greater spreading compared to those on the PLGA scaffold, displaying increased pseudopodial extensions and a more flattened cellular morphology. This observation further confirms that the EGCG/Si coating, by enhancing surface roughness and hydrophilicity, creates a more favorable microenvironment for cell adhesion.

We subsequently evaluated the osteogenic activity of the coatings. Gene expression analysis demonstrated the potent stimulatory effect of the EGCG/Si coating on BMSC differentiation (Figure 3C). Specifically, the coated scaffolds markedly enhanced the mRNA levels of all examined osteogenic markers—*ALP*, *COL1*, *OCN*, *OPN*, *Runx2* and *Osterix*—compared to the uncoated PLGA scaffold. Furthermore, BMSCs cultured on the nSC2 scaffolds exhibited higher expression levels of these genes than those on the nSC1 scaffolds. The ALP activity assay (Figure 3D) revealed that, after culturing for 14 and 21 days, BMSCs in the nSC2 group exhibited markedly increased ALP activity relative to those in the PLGA and nSC1 groups. Moreover, ARS staining (Figure 3E) revealed a similar trend. After 21 days, the PLGA group displayed only sparse mineralized nodules, whereas the EGCG/Si-coated groups showed markedly increased nodule formation, with the nSC2 group exhibiting the most extensive mineralization. Overall, these findings demonstrate a concentration-dependent effect of the EGCG/Si coating on promoting BMSC osteogenic differentiation, where higher surface Si content (as in nSC2 scaffolds) exhibits greater osteoinductive potential. The scaffold’s regulatory influence on BMSCs can be explained by two key factors. Firstly, enhanced surface roughness improves cell adhesion and spreading, potentially triggering osteogenic differentiation through the activation of cytoskeletal tension [34]. Secondly, the released Si ions contribute to osteoinduction by promoting the osteogenic commitment of BMSCs [35].

Angiogenesis constitutes a pivotal phase in tissue regeneration and is inextricably linked to osteogenesis and bone remodeling [36]. This coupling is manifested when nascent blood vessels are recruited to the ossification front, an event that orchestrates the commencement of bone formation and the subsequent reconstruction of bone tissue. To further investigate the pro-angiogenic effects of Si ions, we conducted cell scratch wound and tube formation assays. Firstly, conditioned media were obtained from EGCG/Si-coated scaffolds and applied to HUVEC cultures. The scratch wound assay demonstrated that with prolonged incubation time, cells in all groups exhibited a trend of migration toward the central wound area (Figure 3G). The cell migration rate in the coated scaffold groups was significantly increased compared to the control group, with the nSC2 group showing a more pronounced enhancement than the nSC1 group (Figure 3H). In the tube formation assay, after seeding HUVECs onto Matrigel and culturing for 8 h, tubular networks of varying completeness were observed in all the groups (Figure 3I). Quantitative analysis of key morphological parameters (Figure S3) indicated that, relative to the PLGA group, HUVECs exposed to extracts from the coated scaffolds developed tubular networks exhibiting a higher number of nodes and meshes, along with greater total tube length and mesh area. Notably, the nSC2 group showed statistically significant improvements relative to the PLGA group. In conclusion, these findings indicate that Si ions released from the coatings can effectively enhance HUVEC migration and *in vitro* tube formation capability. This observation aligns with previous reports that Si ions can promote angiogenic functions in HUVECs [37].

### Gene expression profile

**Figure 4:**
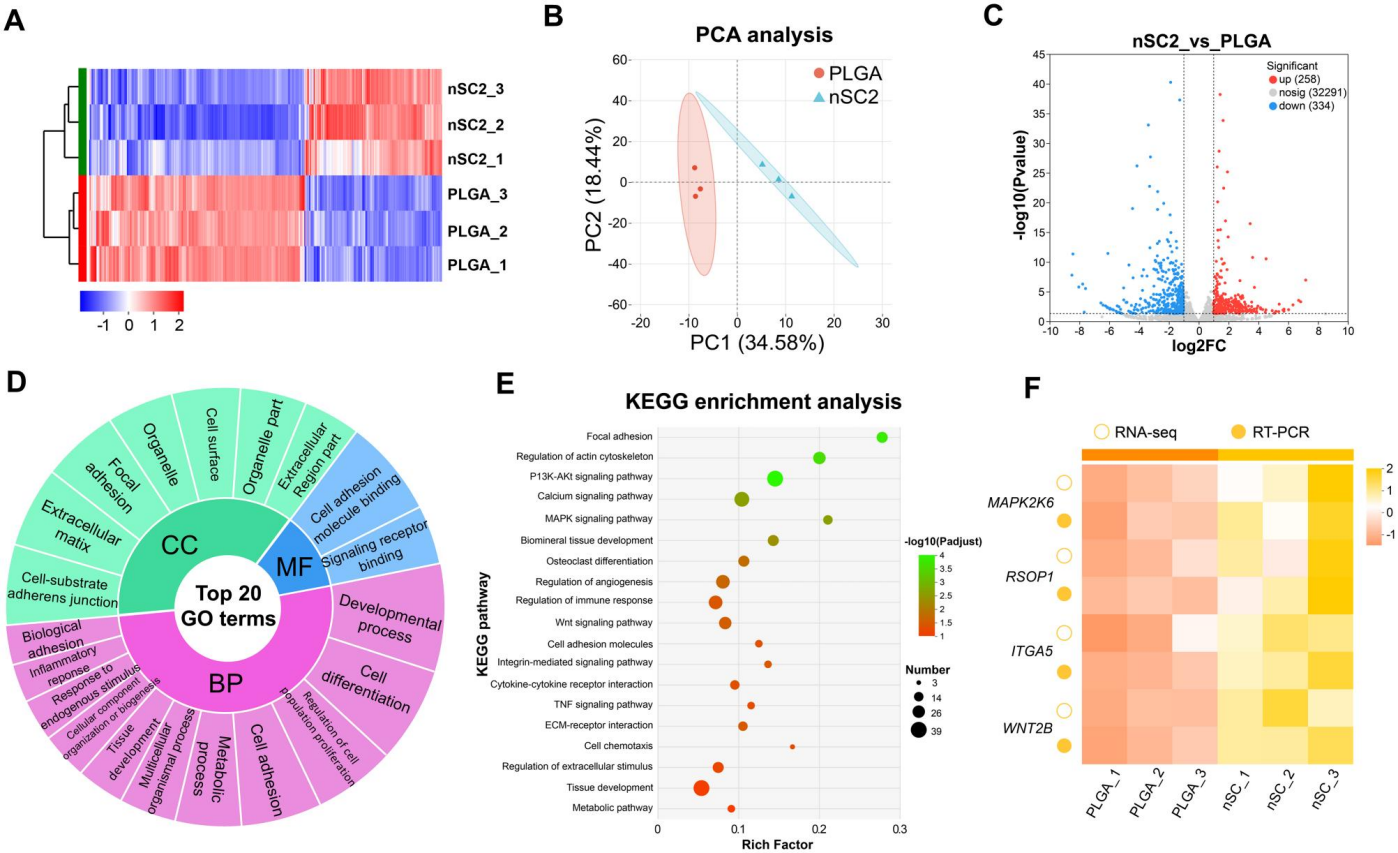
Transcriptomic analysis of BMSCs cultured on scaffold surfaces. (**A**) Heatmap of DEGs. (**B**) PCA analysis of all samples. (**C**) Volcano plot of transcriptomic analysis of DEGs. (**D**) The GO enrichment of the nSC2 group vs the PLGA group. (**E**) The enriched KEGG pathway analysis of the nSC2 group vs the PLGA group. (**F**) Validation of transcriptome data (upregulated 4 DEGs) by RT-PCR.

To elucidate the molecular mechanisms involved in the osteogenic differentiation promoted by the EGCG/Si coating, we conducted transcriptomic analysis of BMSCs cultured on nSC2 scaffolds. The nanohybrid coating markedly altered BMSC gene expression (Figure 4A). Principal component analysis revealed clear segregation between the PLGA and nSC2 groups (Figure 4B). As depicted in the volcano plot (Figure 4C), 258 genes exhibited significant upregulation and 334 showed down-regulation, indicating substantial alterations in the transcriptional profile. These DEGs were further analysed through GO enrichment, classifying them into biological processes, cellular components and molecular functions. The findings emphasized their prominent roles in critical cellular behaviors and developmental processes, such as cell adhesion molecule binding, cell differentiation, tissue development and the regulation of cell population proliferation (Figure 4D).

Subsequently, KEGG pathway analysis was conducted to decipher the underlying signaling mechanisms. The top 20 enriched pathways are shown in Figure 4E. The results revealed a significant enrichment of pathways implicated in cell adhesion, proliferation and osteoblastic differentiation. These pathways included focal adhesion and ECM–receptor interactions, calcium signaling, as well as MAPK and PI3K/Akt signaling pathway. MAPK signaling pathways are critical for cell proliferation [35]. Calcium is an essential component of bone tissue, and the calcium signaling pathway serves as a key regulator in maintaining bone homeostasis [38]. The PI3K/Akt signaling pathway is a crucial lipid kinase cascade that orchestrates the osteogenic differentiation, proliferation and apoptosis of mesenchymal stem cells. Activation of Akt through phosphorylation upregulates the expression of Runx2, a central transcription factor that promotes the transcription of genes critical for bone regeneration and structural remodeling [39]. The Wnt signaling pathways are essential in regulating the osteogenic differentiation of BMSCs [40]. To experimentally validate the transcriptomic findings, we selected four upregulated (*MAPK2K6*, *RSPO1*, *WNT2B* and *ITGA5*) implicated in the aforementioned signaling pathways for RT-qPCR analysis (Figure 4F). The analytical results corroborated that their expression trends agreed well with the transcriptomic findings. Thus, the surface topography and chemical cues generated by the EGCG/Si nanohybrid coating collectively modulate various signaling pathways, ultimately forming a regulatory network that promotes BMSCs adhesion, proliferation and osteogenic differentiation of BMSCs.

### Nanohybrid EGCG/Si coating scaffolds enhance bone regeneration *in vivo*

Given that the implantation of biomaterials *in vivo* inevitably elicits immune responses, the intensity of the stress response induced by implanted materials in immune cells plays a critical role in material-mediated tissue repair [41]. Therefore, the PLGA and nSC2 scaffolds were first subcutaneously implanted in rats to evaluate the foreign body reaction. Histological analysis using H&E staining (Figure S4A) revealed no significant inflammatory response throughout the implantation period in either the PLGA or nSC2 groups, indicating favorable biocompatibility. Meanwhile, neovascularization, as indicated by red arrows, was observed in the nSC2 group at 1, 2 and 4 weeks post-implantation. Immunohistochemical staining for the vascular marker CD31 further substantiated nascent blood vessel formation in the nSC2 group, providing direct histological evidence of enhanced angiogenesis (Figure S4B).

**Figure 5:**
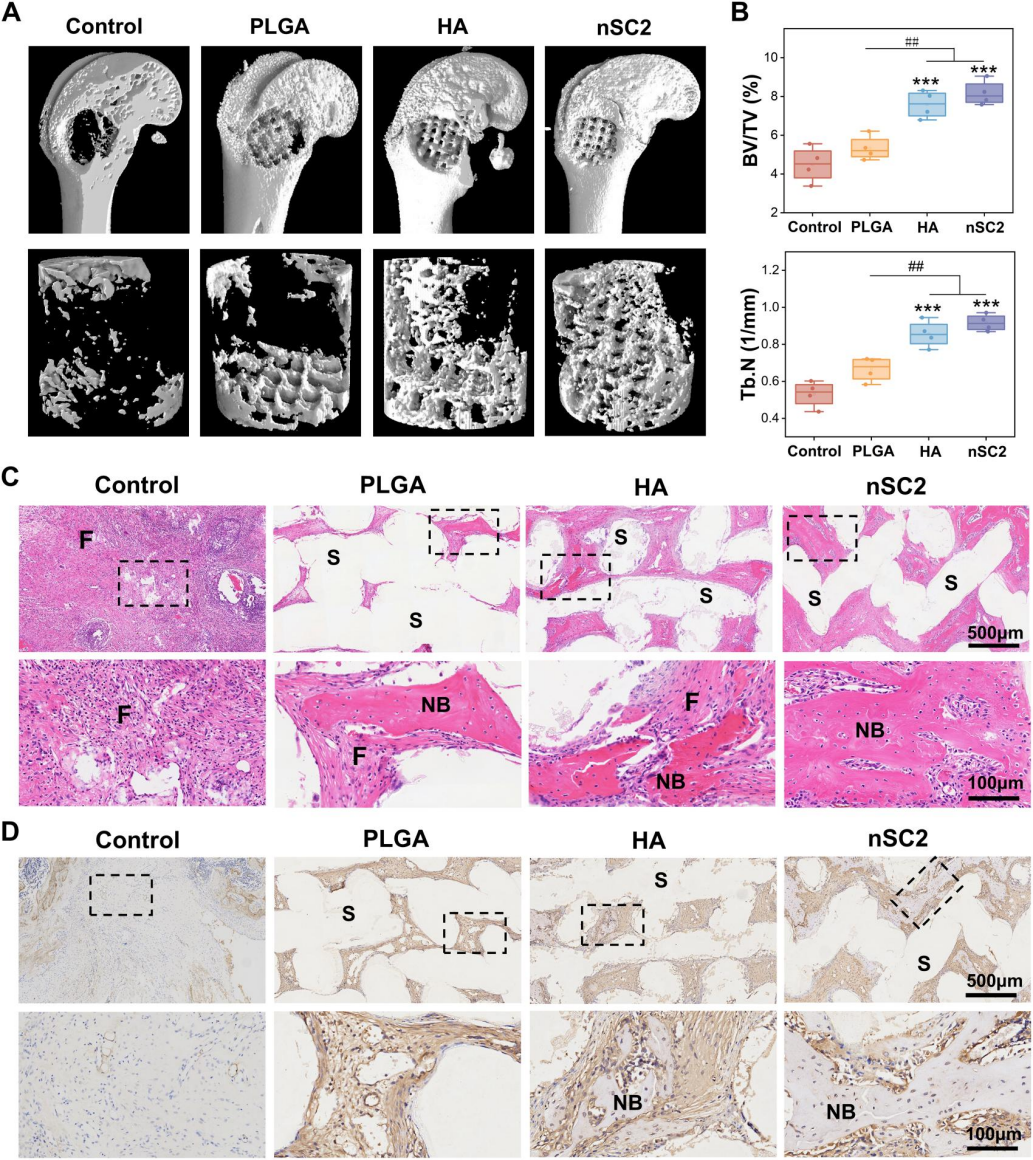
Micro-CT evaluation of scaffold-mediated bone regeneration at week 4. (**A**) Representative micro-CT images showing the femoral condyle defect site in rats. (**B**) Quantitative analysis of BV/TV and Tb.N at the defect site. Representative histological images of H&E staining (**C**) and OCN immunohistochemical staining (**D**) of the defect area four weeks post-implantation. S, scaffolds; F, fibrous tissue; NB, new bone. ***P < 0.001 vs control. ##*P *< 0.01 vs PLGA.

A rat femoral condyle defect model was created to further assess their *in vivo* bone regeneration capacity, with a hydroxyapatite-doped PLGA scaffold serving as the control. Hydroxyapatite is widely regarded as an ideal bone repair implant material due to its chemical and structural similarity to the mineral phase of natural bone and its outstanding osteoconductive properties [42]. Therefore, in this study, HA (nanoscale acicular morphology, see Figure S5) was combined with PLGA to fabricate an organic/inorganic composite material (HA group) for comparative evaluation of the bone regeneration capacity of the nSC2 scaffold. Micro-CT imaging (Figure 5A) revealed new bone tissue formation in all three scaffold groups, extending from the scaffold edges toward the center. The nSC2 group displayed the most extensive new bone formation, whereas the control group predominantly showed empty cavities in the defect area. Bone morphometric analysis (Figure 5B) revealed that the nSC2 group achieved a BV/TV of 8.1 ± 0.6%, which was significantly higher than that of the control (4.5 ± 0.9%) and PLGA (5.3 ± 0.6%) groups. Notably, the nSC2 group exhibited a slight upward trend compared to the HA group (7.6 ± 0.7%), although this difference did not reach statistical significance. Additionally, the nSC2 group displayed the highest Tb.N and the lowest Tb.Sp values (Figure S6), indicative of a denser and more interconnected trabecular microstructure.

Histological section analysis was performed to evaluate the morphological characteristics of the newly formed bone. H&E staining results (Figure 5C) revealed that the defect region in the control group was predominantly filled with fibrous tissue, exhibiting minimal or no new bone formation. In comparison, substantial new bone formation was evident along the implants in the HA and nSC2 groups, while the PLGA group showed only limited bone formation, with most areas occupied by fibrous connective tissue. To further investigate the *in vivo* immunomodulatory activity of the nSC2 scaffold, immunofluorescence staining for iNOS and CD206 was conducted to profile the macrophage polarization state within the defect site. As illustrated in Figure S7A–D, the nSC2 group induced a distinct ‘pro-healing’ immune microenvironment. Specifically, the expression of the pro-inflammatory marker iNOS was significantly suppressed in the nSC2 group compared to the other groups. Conversely, the expression of the reparative marker CD206 was markedly upregulated, indicating a successful macrophage phenotype switch from M1 to M2 driven by the nSC2 scaffold. Consistent with this favorable immune microenvironment, the nSC2 group also exhibited high expression levels of OCN (Figure 5D) and CD31 (Figure S7E). These findings confirm that the nSC2 scaffold effectively promotes coupled angiogenesis and bone extracellular matrix mineralization, likely facilitated by the M2-dominant immunomodulatory niche.

The *in vivo* results confirm that the nSC2 scaffold achieves bone repair efficacy comparable to the gold-standard HA benchmark, exhibiting a trend of superior bone volume and microstructural parameters relative to the control group. This robust regenerative performance stems from the material’s multifaceted modulation of the osteogenic microenvironment. Initially, the immunomodulatory capacity of EGCG plays a pivotal role by driving the polarization of macrophages from a pro-inflammatory M1 phenotype toward a reparative M2 state. These M2 macrophages subsequently secrete paracrine growth factors, thereby establishing an immune microenvironment conducive to bone regeneration [43]. Crucially, our transcriptomic analysis elucidates the molecular mechanisms underlying these cell–material interactions. Regarding physical cues, the EGCG/Si hybrid coating—fabricated via phenol-amine chemistry and electrostatic self-assembly—possesses a unique nanotopography. The significant enrichment of ‘Focal adhesion’ and ‘ECM–receptor interaction’ pathways, alongside *ITGA5* upregulation, provides molecular evidence that this topographical feature enhances integrin-mediated adhesion. This physical interaction likely modulates protein adsorption conformation and strengthens the cell–material interface, effectively ‘priming’ cells for subsequent signaling [44]. Complementing these physical cues, the sustained release of Si ions acts as a chemical stimulus, promoting the secretion of chemokines such as SDF-1 to enhance endogenous BMSC recruitment [45]. Furthermore, the released Si ions activate critical intracellular signaling cascades—including Ca^2+^, Wnt and PI3K-Akt pathways—which cooperatively upregulate osteogenic and angiogenic gene expression [35, 46]. This molecular activation is corroborated by histological evidence, including intense immunostaining for OCN and CD31, as well as robust trabecular bone formation. Consequently, this multifunctional nanohybrid EGCG/Si coating functions as a bioactive signaling platform that integrates topographical cues for enhanced adhesion with biochemical signals for immune modulation, osteogenesis and angiogenesis, ultimately realizing efficient bone tissue regeneration.

## Conclusion

In this work, a multifunctional EGCG/Si nanohybrid coating was constructed on a 3D-printed PLGA scaffold via phenol-amine chemistry and electrostatic self-assembly. The results demonstrated that the released Si ions combined with the surface topological structure of the scaffold, which enhanced cell adhesion and promoted the osteogenic differentiation of BMSCs and angiogenic behavior of HUVECs by activating signaling pathways involved in cell adhesion and differentiation. Additionally, the EGCG-based coating provided the scaffold with superior ROS-scavenging capacity and anti-inflammatory properties, while also promoting macrophage polarization toward the pro-repair M2 phenotype, thereby establishing a conducive immunomodulatory environment for bone regeneration. *In vivo* experiments further confirmed the significant efficacy of the coated scaffold in promoting the repair of bone defects. In conclusion, this EGCG/Si nanohybrid coating strategy provides new insights into the design of multifunctional surfaces for biomedical implants.

## Supplementary Material

rbag035_Supplementary_Data

## References

[1] Wang W , Zhang B, Li M, Li J, Zhang C, Han Y, Wang L, Wang K, Zhou C, Liu L, Fan Y, Zhang X. 3D printing of PLA/n-HA composite scaffolds with customized mechanical properties and biological functions for bone tissue engineering. Compos B: Eng 2021;224:109192.

[2] Lu J , Gao Y, Cao C, Wang H, Ruan Y, Qin K, Liu H, Wang Y, Yang P, Liu Y, Ma Y, Yu Z, Wang Y, Zhong Z, Chang F. 3D bioprinted scaffolds for osteochondral regeneration: advancements and applications. Mater Today Bio 2025;32:101834. 10.1016/j.mtbio.2025.101834 PMC12145566 40487176

[3] Sun X , Xu X, Zhao X, Ma J, Wang T, Yue X, Sun X, Li X, Sun X, Zhang W, Zhang K, Zhang D, Zhao X, Jin W, Wang J. Three-dimensional bioprinted scaffolds loaded with multifunctional magnesium-based metal–organic frameworks improve the senescence microenvironment prompting aged bone defect repair. ACS Nano 2025;19:22141–62. 40509557 10.1021/acsnano.5c03023

[4] Liu K , Li W, Chen S, Wen W, Lu L, Liu M, Zhou C, Luo B. The design, fabrication and evaluation of 3D printed gHNTs/gMgO whiskers/PLLA composite scaffold with honeycomb microstructure for bone tissue engineering. Compos B: Eng 2020;192:108001.

[5] Zhu L , Chen S, Liu K, Wen W, Lu L, Ding S, Zhou C, Luo B. 3D poly (L-lactide)/chitosan micro/nano fibrous scaffolds functionalized with quercetin-polydopamine for enhanced osteogenic and anti-inflammatory activities. Chem Eng J 2020;391:123524.

[6] Li W , Zhong J, Wang X, Zhuang W, Yang Y, He P, Alswadeh M, Li C, Li X, Hu N, Ruan C, Sang H. Intelligent responsive zeolitic imidazolate framework-8@copper oxide nanocomposite 3D-printed scaffolds for efficient repair of infected bone defects. ACS Nano 2025;19:35154–80. 41002264 10.1021/acsnano.5c13201

[7] Magri AMP , Fernandes KR, Assis L, Kido HW, Avanzi IR, Medeiros M, Granito RN, Braga FJC, Rennó ACM. Incorporation of collagen and PLGA in bioactive glass: in vivo biological evaluation. Int J Biol Macromol 2019;134:869–81. 31102678 10.1016/j.ijbiomac.2019.05.090

[8] Li CJ , Park J-H, Jin GS, Mandakhbayar N, Yeo D, Lee JH, Lee J-H, Kim HS, Kim H-W. Strontium/silicon/calcium-releasing hierarchically structured 3D-printed scaffolds accelerate osteochondral defect repair. Adv Healthc Mater 2024;13:e2400154. 38647029 10.1002/adhm.202400154

[9] Qi L , Fang X, Yan J, Pan C, Ge W, Wang J, Shen SGF, Lin K, Zhang L. Magnesium-containing bioceramics stimulate exosomal miR-196a-5p secretion to promote senescent osteogenesis through targeting Hoxa7/MAPK signaling axis. Bioact Mater 2024;33:14–29. 38024235 10.1016/j.bioactmat.2023.10.024 PMC10661166

[10] Lu T , Yuan X, Zhang L, He F, Wang X, Ye J. Enhancing osteoinduction and bone regeneration of biphasic calcium phosphate scaffold thought modulating the balance between pro-osteogenesis and anti-osteoclastogenesis by zinc doping. Mater Today Chem 2023;29:101410.

[11] Han Y , Jia X, Yang Y, Guo P, Li C, Zhang Y, Yin L, Jia B, Wang H. Study of bioactive 3D-printed scaffolds incorporating zinc-based MOF for bone defect repair and anti-inflammatory applications. Mater Today Bio 2025;32:101884. 10.1016/j.mtbio.2025.101884 PMC12159229 40510838

[12] Song Y , Hu Q, Liu S, Wang Y, Jia L, Hu X, Huang C, Zhang H. 3D printed biomimetic composite scaffolds with sequential releasing of copper ions and dexamethasone for cascade regulation of angiogenesis and osteogenesis. Chem Eng J 2024;496:153662.

[13] Arcos D , Vallet-Regí M. Sol–gel silica-based biomaterials and bone tissue regeneration. Acta Biomater 2010;6:2874–88. 20152946 10.1016/j.actbio.2010.02.012

[14] Dong C , Wei H, Zhang X, Li Y, Huang L, Wa Q, Luo Y. 3D printed hydrogel/wesselsite-PCL composite scaffold with structural change from core/shell fibers to microchannels for enhanced bone regeneration. Compos B: Eng 2022;246:110264.

[15] Tobin EJ. Recent coating developments for combination devices in orthopedic and dental applications: a literature review. Adv Drug Deliv Rev 2017;112:88–100. 28159606 10.1016/j.addr.2017.01.007

[16] He Z , Luo H, Wang Z, Chen D, Feng Q, Cao X. Injectable and tissue adhesive EGCG-laden hyaluronic acid hydrogel depot for treating oxidative stress and inflammation. Carbohydr Polym 2023;299:120180. 36876795 10.1016/j.carbpol.2022.120180

[17] He X , Gao Y, Wang X, Zhang C, Xia Z, Xu W, Yang H, Tao G, Cai R, Chen J, He Y. Dual-network hydrogel loaded with antler stem cells conditioned medium and EGCG promotes diabetic wound healing through antibacterial, antioxidant, anti-inflammatory, and angiogenesis. Mater Today Bio 2025;31:101612. 10.1016/j.mtbio.2025.101612 PMC11914521 40104648

[18] Domke A , Jakubowski M, Ławniczak Ł, Ratajczak M, Reczkowski J, Patalas A, Voelkel A, Sandomierski M. Titanium implant modification by ZIF-8 and epigallocatechin gallate-improved bioactivity and antibacterial activity. Surf Coat Technol 2024;494:131372.

[19] Song C , Xu S, Chang L, Zhao X, Mei X, Ren X, Chen Z. Preparation of EGCG decorated, injectable extracellular vesicles for cartilage repair in rat arthritis. Regen Biomater 2021;8:rbab067. 34858635 10.1093/rb/rbab067 PMC8634449

[20] Li Y , Cheng X, Zhang X, Ma Z, Deng C, Liu C, Jian X. Biomimetic metal-phenolic network with cyclolactam hydrogel coating on PPENK implant facilitate bone repair. Chem Eng J 2024;486:150234.

[21] Li L , Li Q, Zhang C, Cao Z, Liu C, Luo R, Wang Y, Zeng X, Fu P. Spatiotemporally modulated polyphenol–protein coating for accelerated healing of chronic wounds. J Mater Chem B 2025;13:8918–38. 40587219 10.1039/d5tb01078k

[22] Sun M , Xu C, Wu R, Ke Y, Ding X, Zhao N, Geng W, Sun Y, Duan S, Xu F-J. Bioinspired amine-guided polyphenol coatings for selective bacterial disruption and osseointegration on orthopedic implants. J Am Chem Soc 2025;147:20144–58. 40439262 10.1021/jacs.5c07074

[23] Liu L , Zhang T, Li C, Jiang G, Wang F, Wang L. Regulating surface roughness of electrospun poly(ε-caprolactone)/β-tricalcium phosphate fibers for enhancing bone tissue regeneration. Eur Polym J 2021;143:110201.

[24] Vallet-Regí M , Ruiz-Hernández E. Bioceramics: from bone regeneration to cancer nanomedicine. Adv Mater 2011;23:5177–218. 22009627 10.1002/adma.201101586

[25] Xu YC , Tang YP, Liu LF, Guo ZH, Shao L. Nanocomposite organic solvent nanofiltration membranes by a highly-efficient mussel-inspired co-deposition strategy. J Membr Sci 2017;526:32–42.

[26] Zhao J , Fang C, Zhu Y, He G, Pan F, Jiang Z, Zhang P, Cao X, Wang B. Manipulating the interfacial interactions of composite membranes via a mussel-inspired approach for enhanced separation selectivity. J Mater Chem A 2015;3:19980–8.

[27] Yang H-C , Liao K-J, Huang H, Wu Q-Y, Wan L-S, Xu Z-K. Mussel-inspired modification of a polymer membrane for ultra-high water permeability and oil-in-water emulsion separation. J Mater Chem A Mater 2014;2:10225–30.

[28] Xu Y , Luo Y, Weng Z, Xu H, Zhang W, Li Q, Liu H, Liu L, Wang Y, Liu X, Liao L, Wang X. Microenvironment-responsive metal-phenolic nanozyme release platform with antibacterial, ROS scavenging, and osteogenesis for periodontitis. ACS Nano 2023;17:18732–46. 37768714 10.1021/acsnano.3c01940

[29] Davenport Huyer L , Pascual-Gil S, Wang Y, Mandla S, Yee B, Radisic M. Advanced strategies for modulation of the material–macrophage interface. Adv Funct Mater 2020;30:1909331.

[30] Li J , Jiang X, Li H, Gelinsky M, Gu Z. Tailoring materials for modulation of macrophage fate. Adv Mater 2021;33:2004172. 10.1002/adma.202004172 PMC9245340 33565154

[31] Zhao X , Pei D, Yang Y, Xu K, Yu J, Zhang Y, Zhang Q, He G, Zhang Y, Li A, Cheng Y, Chen X. Chronic diabetic wound treatment: green tea derivative driven smart hydrogels with desired functions for chronic diabetic wound treatment. Adv Funct Mater 2021;31:2170127.

[32] Kim H-Y , Kang H-G, Nam S-Y, Kim H-M, Jeong H-J. Blockade of RANKL/RANK signaling pathway by epigallocatechin gallate alleviates mast cell-mediated inflammatory reactions. Int Immunopharmacol 2020;88:106872. 32769069 10.1016/j.intimp.2020.106872

[33] Khanam N , Mikoryak C, Draper RK, Balkus KJ. Electrospun linear polyethyleneimine scaffolds for cell growth. Acta Biomater 2007;3:1050–9. 17702681 10.1016/j.actbio.2007.06.005

[34] Li Y , Yang L, Hou Y, Zhang Z, Chen M, Wang M, Liu J, Wang J, Zhao Z, Xie C, Lu X. Polydopamine-mediated graphene oxide and nanohydroxyapatite-incorporated conductive scaffold with an immunomodulatory ability accelerates periodontal bone regeneration in diabetes. Bioact Mater 2022;18:213–27. 35387166 10.1016/j.bioactmat.2022.03.021 PMC8961429

[35] Wang S-J , Jiang D, Zhang Z-Z, Chen Y-R, Yang Z-D, Zhang J-Y, Shi J, Wang X, Yu J-K. Biomimetic nanosilica–collagen scaffolds for in situ bone regeneration: toward a cell-free, one-step surgery. Adv Mater 2019;31:1904341. 10.1002/adma.201904341 31621958

[36] Song S , Zhang G, Chen X, Zheng J, Liu X, Wang Y, Chen Z, Wang Y, Song Y, Zhou Q. HIF-1α increases the osteogenic capacity of ADSCs by coupling angiogenesis and osteogenesis via the HIF-1α/VEGF/AKT/mTOR signaling pathway. J Nanobiotechnology 2023;21:257. 37550736 10.1186/s12951-023-02020-z PMC10405507

[37] Duan W , Jin Y, Cui Y, Xi F, Liu X, Wo F, Wu J. A co-delivery platform for synergistic promotion of angiogenesis based on biodegradable, therapeutic and self-reporting luminescent porous silicon microparticles. Biomaterials 2021;272:120772. 33838529 10.1016/j.biomaterials.2021.120772

[38] Sun X , Jiao X, Wang Z, Ma J, Wang T, Zhu D, Li H, Tang L, Li H, Wang C, Li Y, Xu C, Wang J, Gan Y, Jin W. Polydopamine-coated 3D-printed β-tricalcium phosphate scaffolds to promote the adhesion and osteogenesis of BMSCs for bone-defect repair: mRNA transcriptomic sequencing analysis. J Mater Chem B 2023;11:1725–38. 36723218 10.1039/d2tb02280j

[39] Zhu W , Zhou H, Wei Z, Weng X, Li B, Chen S, Engqvist H, Cui S, Xia W. MII-polyphosphate-based wax-like material with osteogenesis and blood occlusion. Bioact Mater 2025;52:564–73. 40607116 10.1016/j.bioactmat.2025.05.028 PMC12219003

[40] Hao L , Huang S, Huang T, Yi D, Wang C, Cheng L, Guan M, Wu J, Chen D, Pan H, Lu WW, Zhao X. Bone targeting miR-26a loaded exosome-mimetics for bone regeneration therapy by activating Wnt signaling pathway. Chem Eng J 2023;471:144594.

[41] Bank RA. Limiting biomaterial fibrosis. Nat Mater 2019;18:781. 31332318 10.1038/s41563-019-0428-y

[42] Wang H , Li Y, Zuo Y, Li J, Ma S, Cheng L. Biocompatibility and osteogenesis of biomimetic nano-hydroxyapatite/polyamide composite scaffolds for bone tissue engineering. Biomaterials 2007;28:3338–48. 17481726 10.1016/j.biomaterials.2007.04.014

[43] Liu X , Chen W, Shao B, Zhang X, Wang Y, Zhang S, Wu W. Mussel patterned with 4D biodegrading elastomer durably recruits regenerative macrophages to promote regeneration of craniofacial bone. Biomaterials 2021;276:120998. 34237507 10.1016/j.biomaterials.2021.120998

[44] Faia-Torres AB , Guimond-Lischer S, Rottmar M, Charnley M, Goren T, Maniura-Weber K, Spencer ND, Reis RL, Textor M, Neves NM. Differential regulation of osteogenic differentiation of stem cells on surface roughness gradients. Biomaterials 2014;35:9023–32. 25106771 10.1016/j.biomaterials.2014.07.015

[45] Sun J-L , Jiao K, Niu L-N, Jiao Y, Song Q, Shen L-J, Tay FR, Chen J-H. Intrafibrillar silicified collagen scaffold modulates monocyte to promote cell homing, angiogenesis and bone regeneration. Biomaterials 2017;113:203–16. 27821306 10.1016/j.biomaterials.2016.10.050

[46] Khandmaa D , El-Fiqi A, Buitrago J, Perez R, Knowles J, Kim H-W. A mini review focused on the proangiogenic role of silicate ions released from silicon-containing biomaterials. J Tissue Eng 2017;8:204173141770733. 10.1177/2041731417707339 PMC5435366 28560015

