# Supplementary material for: A multifunctional EGCG/Si nanohybrid-coated 3D-printed porous scaffold for bone defect repair
Source: Regen Biomater. 2026 Mar 9;13:rbag035. doi: 10.1093/rb/rbag035 (PMC13070653; doi:10.1093/rb/rbag035)
Supplement: rbag035_Supplementary_Data [file rbag035_supplementary_data.docx]

**A Multifunctional EGCG/Si Nanohybrid-Coated 3D-Printed**

**Porous Scaffold for Bone Defect Repair**

Shiqi Xiao ^1,2,#^, Lin Qi ^3,#^, Jiacheng Wei ^4^, Huan Liu ^4^, Sheng Ding ^1^, Ju chen ^1^, Jin Yang ^1^, Hua Lin ^5,^*, Dianxiang Lu ^1,^*

^1^ Clinical Medical College & Affiliated Hospital of Chengdu University, Chengdu University, Chengdu 610081, China

^2^ Research Center for Nano-Biomaterials, Analytical and Testing Center, Sichuan University, Chengdu 610064, China

^3^ Department of Criminal Science and Technology, Sichuan Police College, Luzhou 646000, China.

^4^ School of Clinical Medicine, North Sichuan Medical College, Nanchong 637000, Sichuan, China

^5^ Department of Stomatology, The First Afﬁliated Hospital of Chengdu Medical College, Chengdu 610500, China

*Corresponding authors

E-mail address: [huaxi2011@sina.com](mailto:huaxi2011@sina.com); [ludianxiang@126.com](mailto:ludianxiang@126.com)

^#^ The first two authors contributed equally to this work

**Text S1. Adhesion and proliferation of BMSCs**

The cells were inoculated onto the scaffolds at a density of 7×10³ cells per well in 48-well culture plates, followed by incubation at 37 °C under humidified conditions containing 5% CO₂, with the medium replaced every 48 h. The adhesion of BMSCs on the scaffolds was evaluated using SEM. Prior to imaging, samples were rinsed with PBS, fixed with 2.5 vol% glutaraldehyde, dehydrated through a graded series of tert-butanol, and vacuum-dried at 25 °C. Cell proliferation was analyzed using a CCK-8 (KeyGEN, China) assay after culturing for 1, 3, and 5 days. For this assay, the CCK-8 working solution was introduced into each well and incubated at 37 °C for 2 h. Afterwards, 100 μL of the reaction mixture from each well was transferred to a fresh 96-well plate, and the absorbance was read at 450 nm to evaluate cell viability.

**Text S2. Osteogenesis evaluation of BMSCs**

BMSCs were cultured on scaffolds at a seeding density of 2 × 10⁴ cells per well. The medium was renewed every 48 h until sample collection.

**Alkaline phosphatase (ALP) staining:** ALP activity was evaluated after 7, 14, and 21 days of incubation using an ALP Assay Kit. This assay is based on the enzymatic conversion of p-nitrophenylphosphate to p-nitrophenol, and the generated product was quantified spectrophotometrically at 405 nm with a multilabel plate reader. ALP levels were standardized relative to the total protein content in each sample, which was measured using a Bicinchoninic Acid Protein Assay Kit. All experiments were performed in triplicate to ensure consistency and reliability of results.

**ARS staining:** After 21 days of culture, calcium mineralization was evaluated by ARS staining. Samples were fixed with 95% ethanol, followed by staining with 0.1% (w/v) Alizarin Red S (pH 4.2) for 10 min at RT. After rinsing, mineral deposits were visualized under an optical microscope.

**Osteogenic genes expression analysis**: After 7 days of culture, the expression levels of osteogenic markers (*ALP*, *COL1*, *OCN*, *OPN*, *Osterix*, and *Runx2*) were evaluated using RT-PCR. Relative gene expression was calculated using the 2⁻^ΔΔCt^ method and normalized to the control group, which consisted of cells grown on standard tissue culture plates. The primer sequences utilized in this study are provided in **Table S3**.


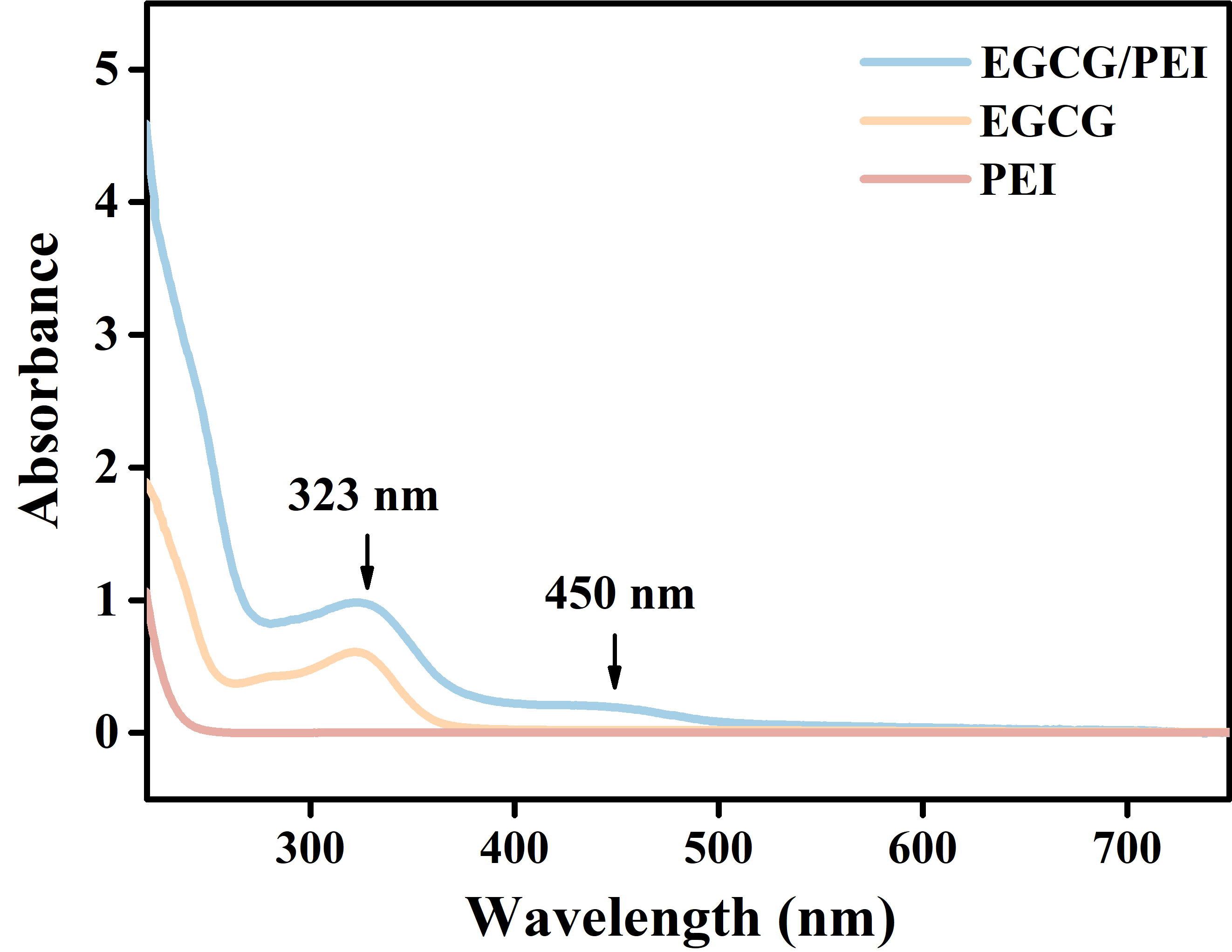


**Fig. S1**. UV–vis spectra of 10-fold diluted EGCG, PEI, and EGCG/PEI solutions (molar ratio of 1:2).


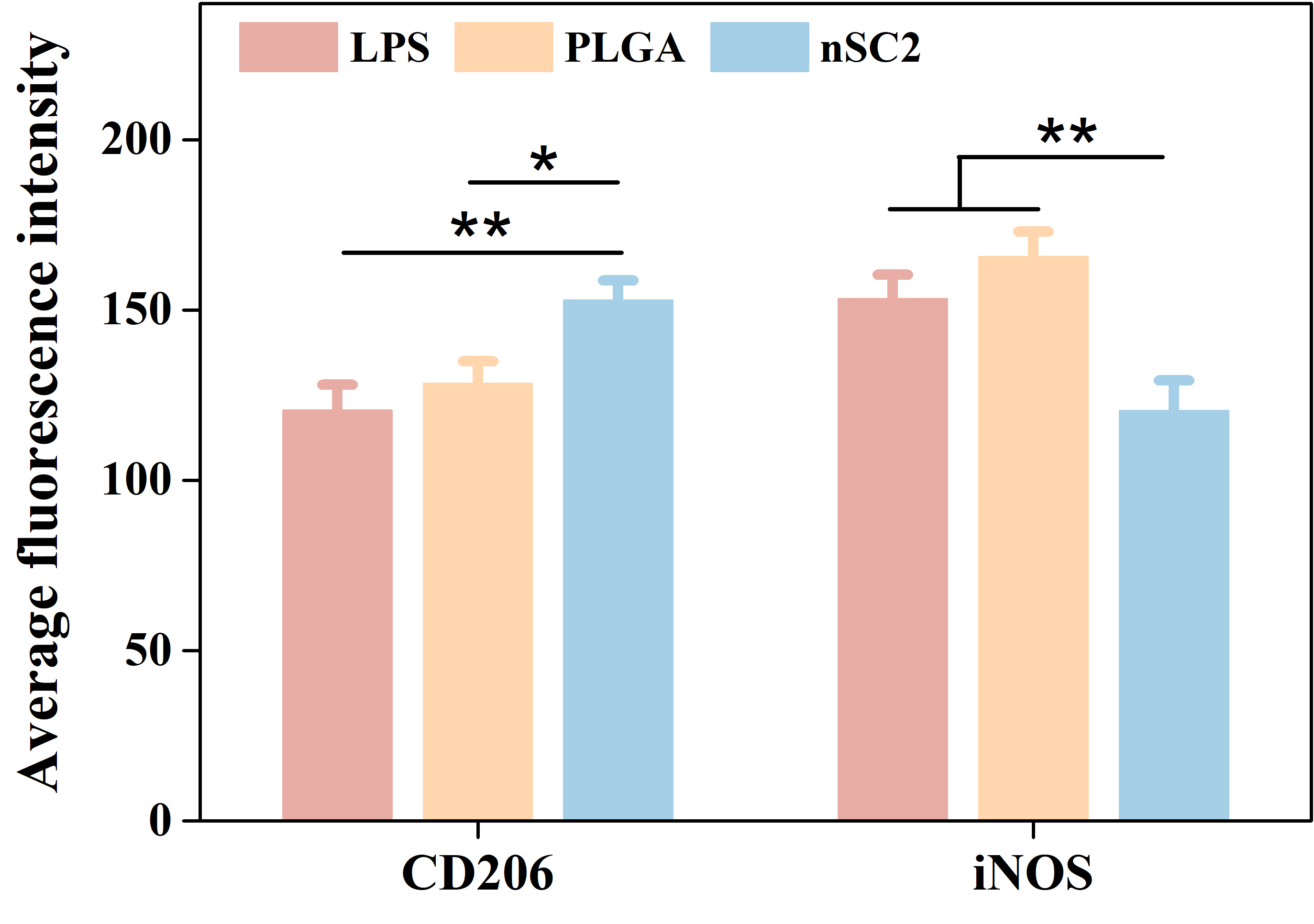


**Fig. S2**. Quantitative analysis of the average fluorescence intensity of CD206 and iNOS. **p* < 0.05, and ***p* < 0.01.


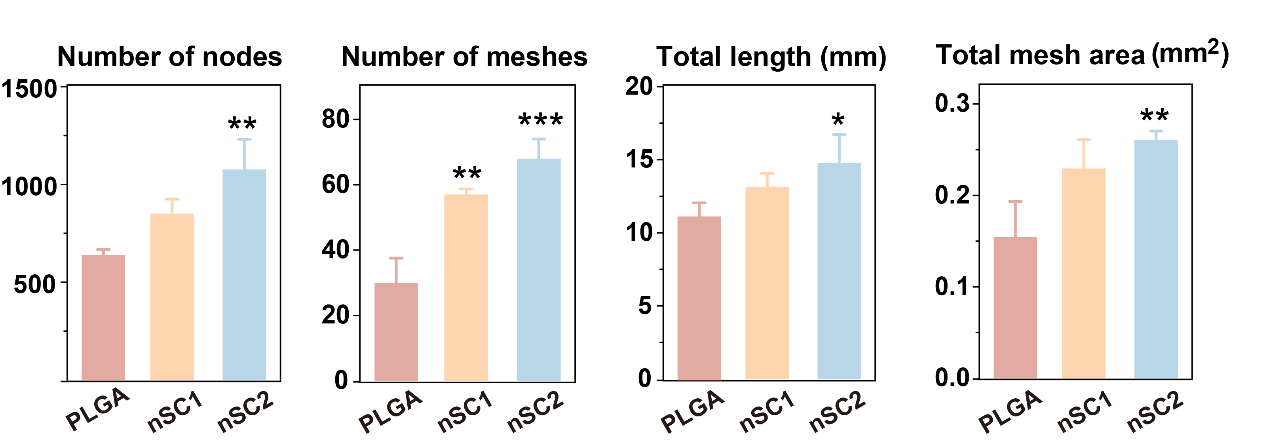


**Fig. S3**. Quantification of meshes, nodes, total length, and total mesh area of the tube. **p* < 0.05, ***p* < 0.01, and ****p* < 0.001 vs control.


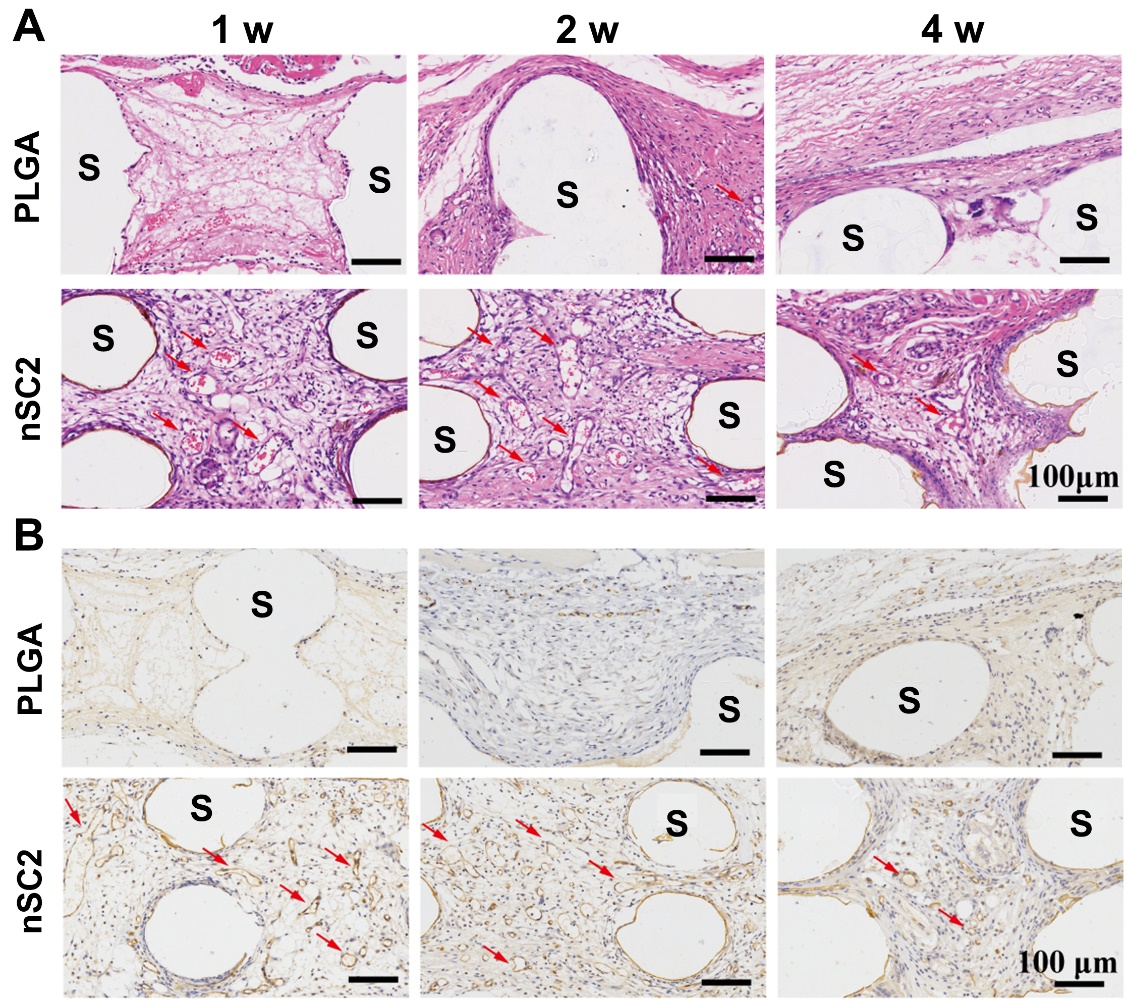


**Fig. S4**. H&E and CD31 staining images of subcutaneous implantation of scaffolds in rats after 1, 2, and 4 w. S indicates scaffold material, the red arrow indicates new vessels.





**Fig. S5**. TEM images of HA.


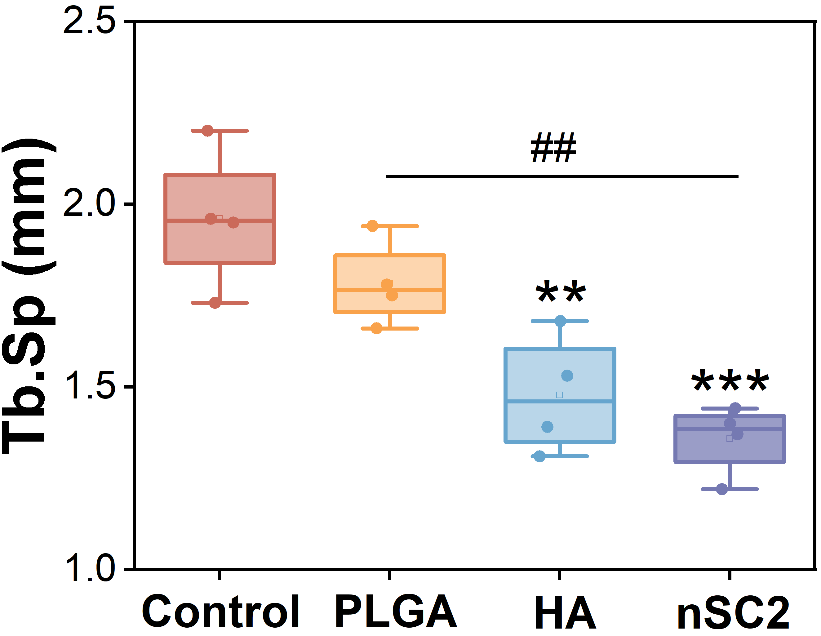


**Fig. S6**. Quantitative analysis of Tb. Sp. ***p* < 0.01 and ****p* < 0.001 vs control.


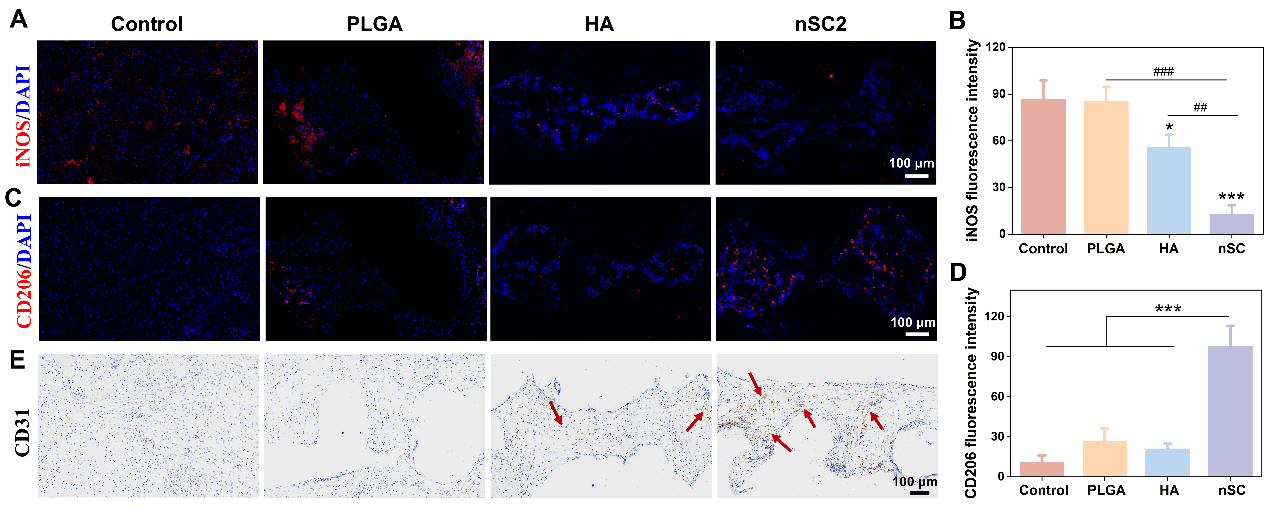


**Fig. S7**. (A–D) Immunofluorescence staining of iNOS and CD206 in defect areas, and fluorescence intensity. (E) CD31 immunohistochemical staining in defect areas. The red arrow indicates new vessels. **p* < 0.05 and ****p* < 0.001 vs control. ##*p* < 0.01 and ###*p* < 0.001 vs nSC.

**Table S1.** The materials used in the experiment and their manufacturers

| Material | Manufacturer |
| --- | --- |
| PLGA | Jinan Daigang Biomaterial Co., Ltd, China |
| EGCG | Sigma-Aldrich Company, USA |
| PEI | Aladdin Co., Ltd, China |
| Tetramethyl orthosilicate | Aladdin Co., Ltd, China |
| HA | Beijing Dekedaojin Technology Co., Ltd, China |
| dichloromethane | Chengdu Kelong Chemical Reagent Co., Ltd, China |
| α-MEM | Gibco, USA |
| NBCS | Gibco, USA |
| penicillin and streptomycin | MP Biomedicals, USA |
| CCK-8 | KeyGEN, China |
| ALP Assay Kit | Beyotime, China |
| ROS Assay Kit | Beyotime, China |
| DPPH | Nanjing Jiancheng Bioengineering Insititute, China |
| Matrigel | BD Biosciences, USA |

**Table S2.** Test instruments used in the experiment and their models

| Instrument | model |
| --- | --- |
| 3D bioprinter | Envision TEC, Germany |
| SEM | JSM-6510LV, JEOL, Japan |
| TEM | Tecnai G2 F20 S-TWIN, USA |
| EDS | X-MaxN 20, Oxford, UK |
| XPS | AXIS Supra, Kratos, UK |
| UV–vis spectrophotometer | UV3600, Shimadzu, Japan |
| Water contact angle measurements | JY-82B, Chengde Dingsheng, China |
| ‌Zeta potential analyzer‌ | SurPASS, Anton Paar, Austria |
| ICP-OES | Agilent 5100 SVDV, USA |
| Multilabel plate reader | PerkinElmer, USA |
| Optical microscope | Nikon DS-Fi2, Japan |
| RT-PCR | QuantStudio ^TM^3, ThermoFisher, USA |
| Confocal laser scanning microscope | Nikon AIR MP+, Japan |
| Illumina NovaSeq 6000 platform | Illumina, USA |
| Micro-CT | VivaCT80, SCANCO Medical AG, Switzerland |

**Table S3.** Primer sequences used in the present study

|  | Forward primers (5’-3’) | Reverse primers (5’-3’) |
| --- | --- | --- |
| *β-actin* | catcactatcggcaatgagcggttcc | acgcagctcagtaacagtccgccta |
| *TNF-α*  *IL-1β*  *IL-10*  *Arg-1* | CCCTCACACTCACAAACCACC  TCAAATCTCGCAGCAGCACATC  AATAAGCTCCAAGACCAAGGTGT  CTGGGGATTGGCAAGGTGAT | CTTTGAGATCCATGCCGTTG  CGTCACACACCAGCAGGTTATC  CATCATGTATGCTTCTATGCAGTTG  CAGCCCGTCGACATCAAAG |
| *ALP* | gcagaagccgccaacctgtg | ctgtcctgagcatcagcatgagtc |
| *COL1* | gcgaacagggcgacagaggcataaag | ggaccaacaggaccagcatcaccagt |
| *OCN* | ggaccctctctctgctcactctg | accttactgccctcctgcttgg |
| *OPN* | aacactcagatgctgtagccacttg | gctttcattggagttgcttggaagag |
| *Osterix* | cggcaaggtgtacggcaagg | gagcagagcagacaggtgaacttc |
| *Runx2* | cggcaagatgagcgacgtgag | tgctgctgctgctgctgttg |
| *MAPK2K6* | GGGCATCACCATGATTGAGTT | GGGAGTGCCCCAAGAATCATA |
| *RSOP1* | CACAAGGGCCGCTGCTAT | ACCACTCGCTCATTTCACATTG |
| *ITGA5* | CTGCTCGGCCTGCTCATC | CGGGAGGGAGCGTTTGA |
| *WNT2B* | CTTGGACAAGGCTGCAGGTT | CGTTCCTTTCGATGTCTTGCT |
